# The Relationship Between Facial Expression and Cognitive Function in Patients With Depression

**DOI:** 10.3389/fpsyg.2021.648346

**Published:** 2021-06-21

**Authors:** Ma Ruihua, Guo Hua, Zhao Meng, Chen Nan, Liu Panqi, Liu Sijia, Shi Jing, Tan Yunlong, Tan Shuping, Yang Fude, Tian Li, Wang Zhiren

**Affiliations:** ^1^Peking University HuiLongGuan Clinical Medical School, Beijing Huilongguan Hospital, Beijing, China; ^2^Zhumadian Psychiatric Hospital, Zhumadian, China; ^3^Department of Neurosurgery, Sanbo Brain Hospital, Capital Medical University, Beijing, China; ^4^Department of Physiology, Faculty of Medicine, Institute of Biomedicine and Translational Medicine, University of Tartu, Tartu, Estonia

**Keywords:** depression, cognitive function, facial expression recognition, processing speed, problem-solving

## Abstract

**Objective:** Considerable evidence has shown that facial expression recognition ability and cognitive function are impaired in patients with depression. We aimed to investigate the relationship between facial expression recognition and cognitive function in patients with depression.

**Methods:** A total of 51 participants (i.e., 31 patients with depression and 20 healthy control subjects) underwent facial expression recognition tests, measuring anger, fear, disgust, sadness, happiness, and surprise. The Chinese version of the MATRICS Consensus Cognitive Battery (MCCB), which assesses seven cognitive domains, was used.

**Results:** When compared with a control group, there were differences in the recognition of the expressions of sadness (*p* = 0.036), happiness (*p* = 0.041), and disgust (*p* = 0.030) in a depression group. In terms of cognitive function, the scores of patients with depression in the Trail Making Test (TMT; *p* < 0.001), symbol coding (*p* < 0.001), spatial span (*p* < 0.001), mazes (*p* = 0.007), the Brief Visuospatial Memory Test (BVMT; *p* = 0.001), category fluency (*p* = 0.029), and continuous performance test (*p* = 0.001) were lower than those of the control group, and the difference was statistically significant. The accuracy of sadness and disgust expression recognition in patients with depression was significantly positively correlated with cognitive function scores. The deficits in sadness expression recognition were significantly correlated with the TMT (*p* = 0.001, *r* = 0.561), symbol coding (*p* = 0.001, *r* = 0.596), maze (*p* = 0.015, *r* = 0.439), and the BVMT (*p* = 0.044, *r* = 0.370). The deficits in disgust expression recognition were significantly correlated with impairments in the TMT (*p* = 0.005, *r* = 0.501) and symbol coding (*p* = 0.001, *r* = 0.560).

**Conclusion:** Since cognitive function is impaired in patients with depression, the ability to recognize negative facial expressions declines, which is mainly reflected in processing speed, reasoning, problem-solving, and memory.

## Introduction

Facial expression recognition isx considered an essential skill for successful social interactions, and it represents how others think of an individual and can potentially provide vague self-referential cues for making inferences and decisions. Accurate recognition of facial expressions is critical for the composition of interpersonal and cognitive functions (Wang et al., 2011; Hiser and Koenigs, 2018). In the 1970s, Ekman and Friesen proposed that human facial expressions contain six basic emotions, namely, happiness, sadness, anger, fear, disgust, and surprise. These basic emotions are stable across cultures and races (Goghari and Sponheim, 2013). The facial emotion recognition deficit is a disorder, in which individuals experience difficulty in recognizing the emotional states of other people through facial expressions. Such a deficit may lead to a misunderstanding of the social behavior of others and interfere with the social function of an individual (Behere, 2015; Lee et al., 2020). The deficits in facial expression recognition have been found to be associated with depression and other mental illnesses (Cotter et al., 2018; Hayashi et al., 2021).

Cognitive theories suggest that people with depression interpret self-referential social information negatively, including facial expression recognition (Bone et al., 2019). Beck (1967) proposed that negative self-patterns may affect the interpretation of facial expressions, for example, by making ambiguous expressions more susceptible to negative interpretations. Since then, some studies have found that depressed people have a reduced ability to recognize facial expressions, in which neutral or ambiguous faces may be interpreted as sad (Gur et al., 1992; Gollan et al., 2010; Lee et al., 2016). In recent studies of depression, negative processing bias is considered to play a causal role in the development of depressive symptoms (Roiser et al., 2012). Studies have found that when compared with healthy controls, patients with depression are less accurate in recognizing happy expressions or are less likely to interpret neutral faces as happiness (Zwick and Wolkenstein, 2016; Vidal-Ribas et al., 2018). However, the results of the studies on facial expression recognition in patients with depression remain mixed.

Facial expression recognition is a special field of cognitive function. Impaired facial affect recognition and cognitive deficits are prevalent in people with depression (Vechetova et al., 2019). Being a core symptom of depression, cognitive dysfunction may be the underlying phenotype of affective disorders (Miskowiak et al., 2016). The degree of cognitive impairment and the severity of disease were found to be associated with deficits in facial expression recognition (Wiechetek Ostos et al., 2011; Sheardova et al., 2014). The deficits in facial expression recognition are related to cognitive functions such as working memory, attention, and even language ability (Barrett et al., 2011; Heck et al., 2017; Leib, 2019). Some studies have found that executive functions are related to deficits in facial expression recognition among adults with mental illnesses, such as schizophrenia (Yang et al., 2015). Similarly, Phillips et al. (2010) found that executive function could predict the performance of facial expression recognition in patients with depression. Research on cognitive bias suggests that depression is characterized by negative automatic thinking and biases in attention, interpretation, and memory (Mathews and MacLeod, 2005). The extant literature indicates that cognitive deficits in memory, processing speed, and executive functioning are particularly common in depression (Woo et al., 2016; Rao et al., 2019). However, the relationship between facial expression recognition and various aspects of cognitive impairment resulting from depression is yet to be fully determined. Accordingly, research on potential mechanisms, such as deficits in the recognition of emotions of other people, which might link the impaired cognitive function to interpersonal problems and behavioral disturbances, would increase our understanding of life with cognitive impairment in depression. The tests of emotion recognition may represent a potential tool for detecting early-stage cognitive impairment. Therefore, this study aimed to explore the relationship between facial expression recognition and cognitive function in patients with depression.

## Materials and Methods

### Ethics Statement

This study was reviewed and approved by the Ethics Committee of Beijing Huilongguan Hospital and the Ethics Committee of Zhumadian Psychiatric Hospital (ethics approval number: 2016-72). All subjects were informed of the content before the trial, freely volunteered to participate, and signed an informed consent form.

### Participants

A total of 51 participants took part in this study, including 31 patients with depression and 20 healthy individuals, with similar age, gender, and education.

In the patient group, we recruited patients with depression who were outpatients or inpatients at Zhumadian Psychiatric Hospital from July 2018 to August 2019. The inclusion criteria were as follows: (1) met the United States Diagnostic and Statistical Manual, Fourth Edition (DSM-IV) criteria for depression diagnosis, (2) the Hamilton Depression Rating Scale (HAMD-17) score of ≥17 points, with more than two depressive episodes, (3) Han nationality, and (4) aged 18–50 years. The exclusion criteria were as follows: (1) presence of other mental disorder (comorbid anxiety disorders were not excluded), (2) history of cerebral organic diseases, cerebral injury, electrical shock treatment, or other serious physical diseases, (3) history of alcohol and substance abuse, (4) mental retardation, (5) pregnant and lactating women, and (6) claustrophobia.

In the control group, healthy subjects in the community surrounding the Zhumadian Second People’s Hospital during the same period were enrolled. The inclusion criteria were as follows: (1) no past history of mental disorders, (2) matched the race, age, sex, and years of education of the patient groups, (3) HAMD-17 score of <7 points, and (4) no family history of mental disorders. The exclusion criteria were as follows: (1) first-degree relatives diagnosed with a mental illness, (2) history of cerebral organic diseases, cerebral injury, electrical shock treatment, or other serious physical illnesses, (3) history of alcohol and substance abuse, (4) mental retardation, and (5) pregnant and lactating women.

### Facial Expression Recognition

**Figure 1:**
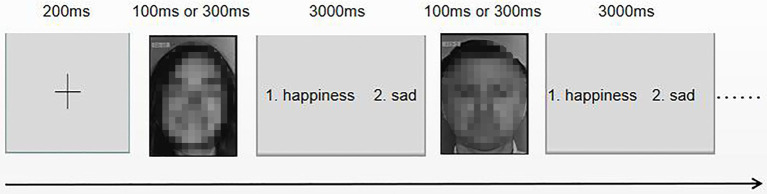
Flowchart of facial expression recognition.

The participants performed the facial expression recognition tests. Ten models (i.e., six females and four males) were selected from the Ekman gallery. Each model had six unique facial expressions (i.e., happiness, sadness, fear, disgust, surprise, and anger), with a total of 60 pictures. The experimental program was compiled and run with E-prime 2.0 (experimental program software). Before the test, the participants were informed about the rules of the test and practiced. In each trial, the participants were asked to distinguish between the two facial expressions of 10 models (i.e., a total of 15 sets of faces, each set of 20 pictures). As shown in Figure 1, after the subjects pressed the “space” key, the participants were presented with a fixation point “+” for 200 ms in the center of the screen. Subsequently, the fixation point disappeared, and then a picture of the emotional expression of the model was randomly presented. The presentation time was randomly set for the picture to appear for either 100 or 300 ms. The task was to choose one or two of the two expression options to judge the expression presented. To prevent the subjects from stopping the task or being distracted during the task, they were asked to make a judgment within 3,000 ms; otherwise, the task automatically jumped to the next picture. An expression without a judgment was marked as incorrect. The advantage of this paradigm is that it is a comprehensive test of basic facial recognition.

### Cognitive Function Test

All participants were instructed to complete the MATRICS Consensus Cognitive Battery (MCCB) using a computer under the guidance of an experienced physician. The 10 subtests of the MCCB were organized into the following seven domains:

1. Speed of processing (SOP): Trail Making Test (TMT), Brief Assessment of Cognition in Schizophrenia (BACS) symbol coding (SC), and category fluency (CF)
2. Attention/vigilance (AV): continuous performance test-identical pairs (CPT-IP)
3. Working memory (WM): spatial span (SS) and digital sequence (DS)
4. Verbal learning (VBL): Hopkins Verbal Learning Test-Revised (HVLT-R)
5. Visual learning (VSL): Brief Visuospatial Memory Rest-Revised (BVMT-R)
6. Reasoning and problem-solving (RPS): Neuropsychological Assessment Battery (NAB) mazes
7. Social cognition (SC): Mayer-Salovey-Caruso Emotional Intelligence Test (MSCEIT) managing emotions (ME).

### Statistical Analysis

The statistical analysis was performed using IBM SPSS Statistics (SPSS Inc., Chicago, Illinois, USA, Version 23.0). The experimental data of each group were expressed as *x* ± *s*. The chi-square test was used for sex between groups, and an independent sample *t*-test was used to compare age and education level. For the bilateral test, *p* < 0.05 was considered statistically significant.

We used to represent the accuracy of facial expression recognition – the measurement value of discriminate ability – which follows the signal detection theory and uses the hit rate and false positive rate to estimate recognition ability (Macmillan and Creelman, 2005). Raw scores for each of the 10 MCCB tests were transformed into *T*-scores. First, we calculated a series of *t*-tests to assess differences between patient groups and controls regarding emotion recognition performance and cognitive function variables. Second, we assessed the bivariate two-tailed Pearson’s correlations between facial expression recognition and cognitive scores in patients with depression. Finally, we used Pearson’s correlation to analyze the relationship between facial expressions that were significantly associated with cognitive function scores and various cognitive domains.

## Results

Descriptive information is presented in Table 1. There were no significant differences in age (*F* = 5.71, *p* = 0.87), gender (*F* = 1.07, *p* = 0.30), or years of education (*F* = 0.23, *p* = 0.09).

**Table 1:** Descriptive information.

	Depression (*n* = 31)	Health control (*n* = 20)	*t*/*F*/*χ*^2^	*p*
Age (year, *x* ± *s*)^*^	26.71 ± 9.30	27.10 ± 6.32	5.71	0.87
Gender (case, male/female)^&^	14/17	12/8	1.07	0.30
Years of education (year, *x* ± *s*)^*^	10.77 ± 2.90	12.15 ± 2.48	0.23	0.09
HAMD-17 (score,^−^*x* ± *s*)	21.71 ± 4.42	-	1.75	0.72
HAMA (score)	22.74 ± 9.54	-	1.07	0.72

* Represents independent sample *t*-test.

& Represents chi-square test.

**Figure 2:**
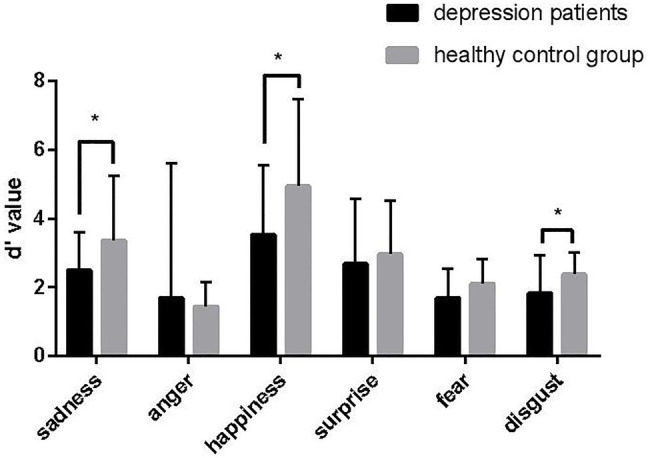
Accuracy of facial expression recognition (mean ± SD). *Represent for p < 0.05.

As shown in Table 2 and Figure 2, compared with the healthy control group, there were differences in the recognition of sadness (*p* = 0.036), happiness (*p* = 0.041), and disgust (*p* = 0.030) expressions in the depression group.

**Table 2:** Facial expression recognition between depression group and health control group.

	Depression group	Health control	*p*
Sadness^*^	2.50 ± 1.09	3.37 ± 1.89	0.036
Anger	1.70 ± 3.90	1.46 ± 0.70	0.794
Happiness^*^	3.53 ± 2.03	4.95 ± 2.51	0.041
Surprise	2.69 ± 1.87	2.97 ± 1.55	0.585
Fear	1.70 ± 0.84	2.11 ± 0.70	0.080
Disgust^*^	1.84 ± 1.10	2.38 ± 0.63	0.030

* Represent for p < 0.05.

**Figure 3:**
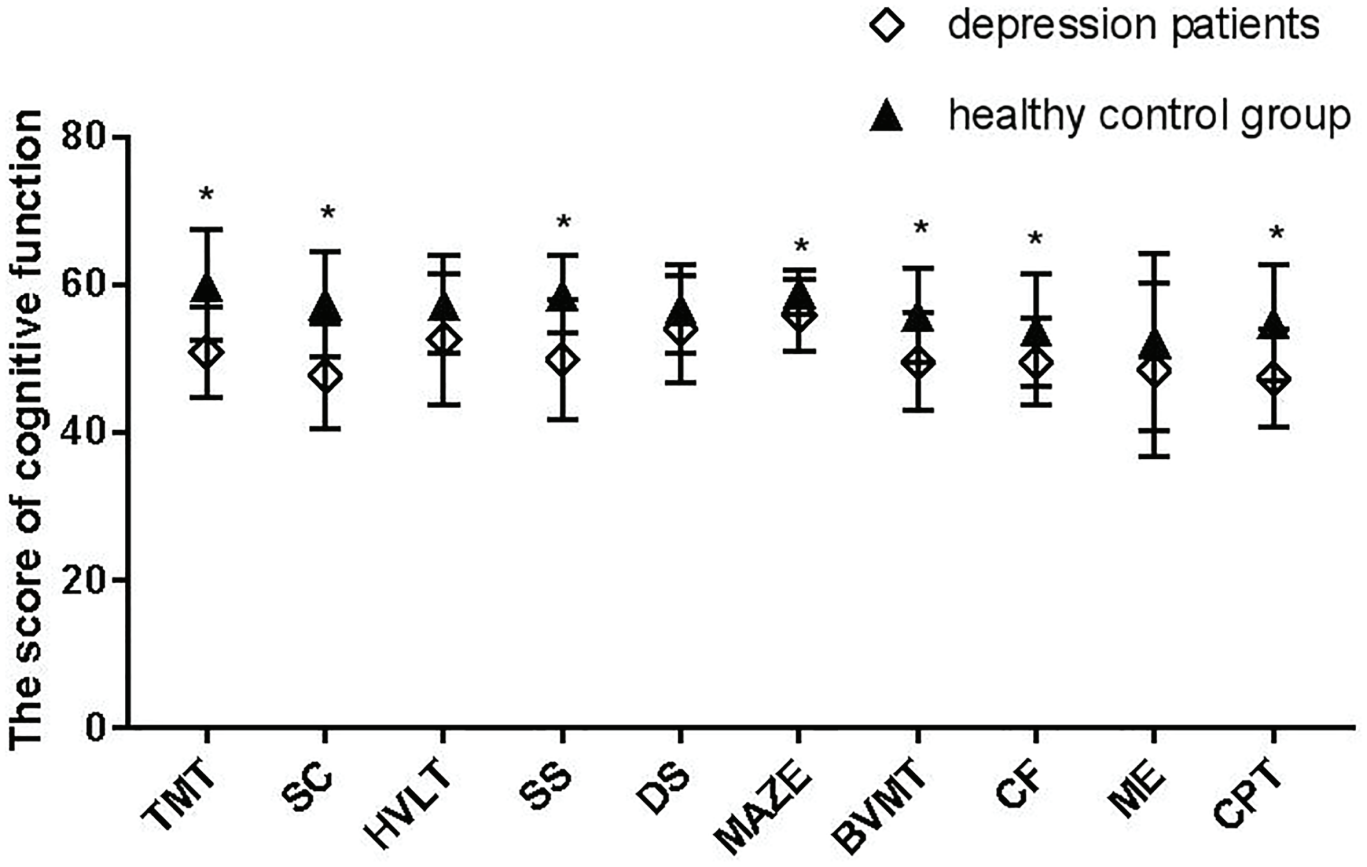
MATRICS Consensus Cognitive Battery (MCCB) scores for the depression group and control group. *Represent for p < 0.05.

There are significant differences in TMT (*p* < 0.001), SC (*p* < 0.001), SS (*p* < 0.001), MAZE (*p* = 0.007), BVMT (*p* = 0.001), CF (*p* = 0.029), and CPT (*p* = 0.001) between the depression group and the control group (Figure 3).

**Figure 4:**
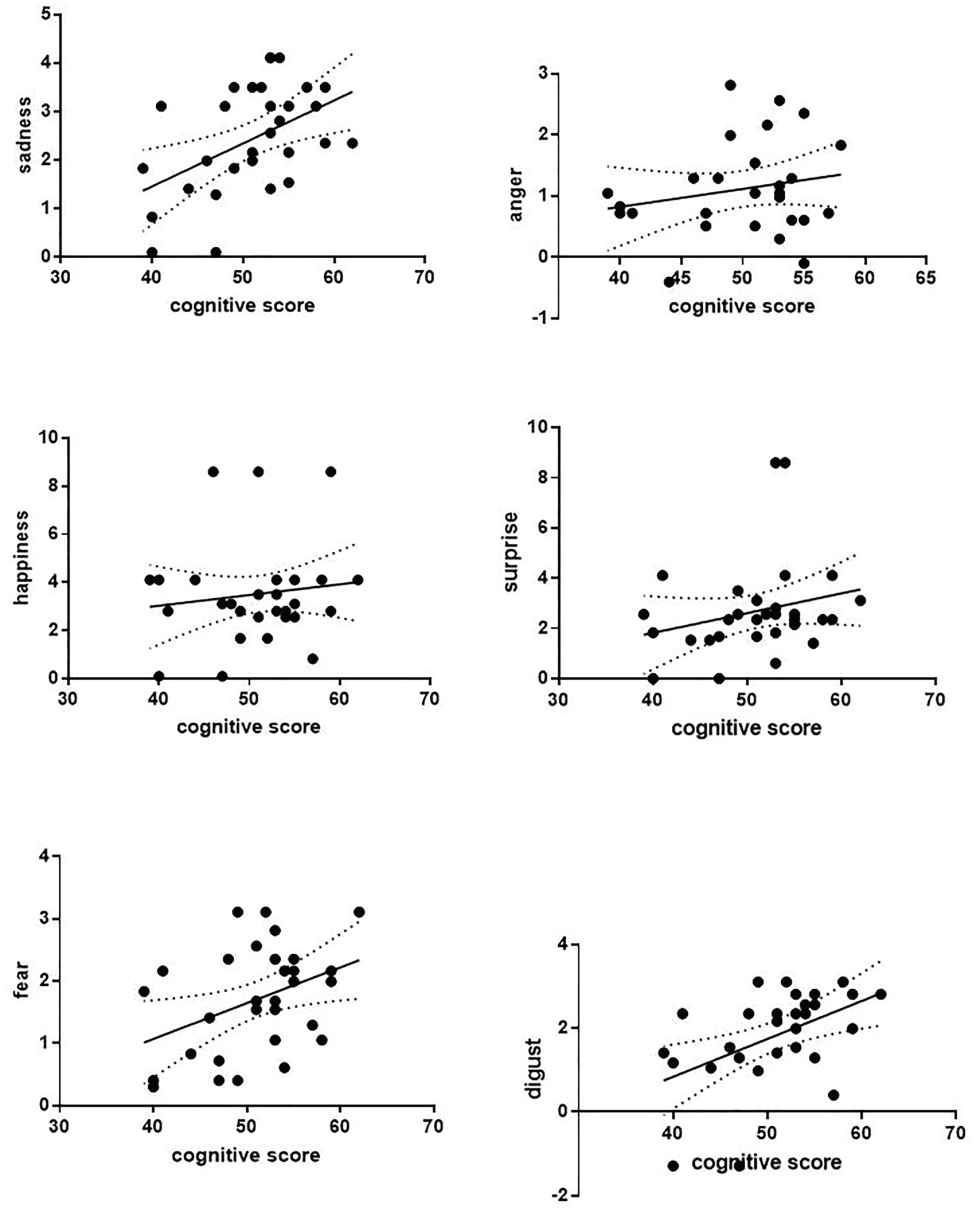
Correlation relationship between facial expression recognition and cognitive score.

**Figure 5:**
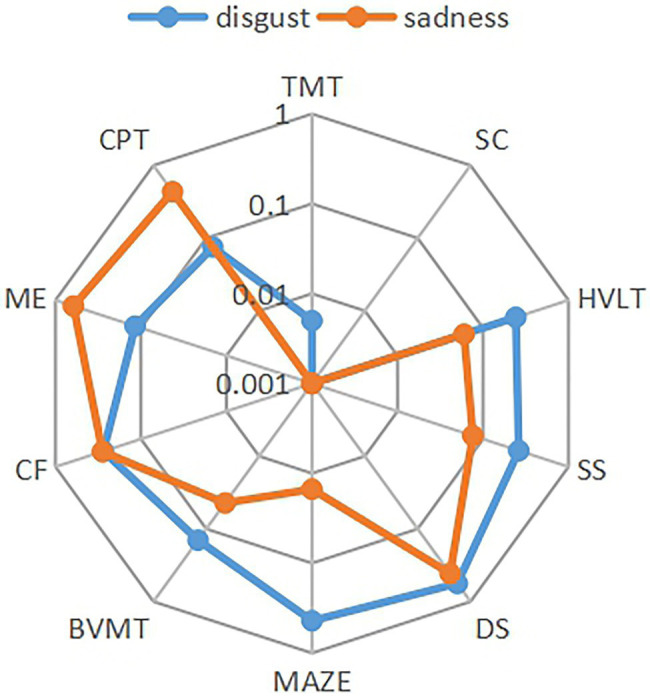
Relationship between the expressions of disgust and sadness and cognitive domain.

The correlation analysis of the relationship between six facial expressions and total cognitive scores was performed. Except for the weak correlation between disgust expression and cognitive score (*p* = 0.049, *r* = 0.445), other facial expressions were not correlated with cognitive scores in the control group. It also shows that the recognition of sadness (*p* = 0.005, *r* = 0.502) and disgust (*p* = 0.006, *r* = 0.486) facial expressions was positively correlated with cognitive function in patients with depression (Figure 4). The correlation analysis was performed between facial expression recognition and the cognitive domain in the depression group (Figure 5). Recognition of sadness facial expressions was related to TMT (*p* = 0.001, *r* = 0.561), SC (*p* = 0.001, *r* = 0.596), MAZE (*p* = 0.015, *r* = 0.439), and BVMT (*p* = 0.044, *r* = 0.370). Recognition of disgust facial expressions was related to TMT (*p* = 0.005, *r* = 0.501) and SC (*p* = 0.001, *r* = 0.560).

## Discussion

In this study, we examined the association between cognitive function and recognition of emotions in facial expressions for patients with depression. The main finding was that people with depression were less able to recognize facial expressions, especially in differentiating negative expressions. Earlier studies found a negative bias in facial expression recognition for depression, which leads to a tendency to identify neutral faces as sad ones (Gur et al., 1992; Leppänen et al., 2004; Lee et al., 2016). Our study further found that people with depression have trouble distinguishing between negative and neutral expressions. This difference may be due to differences in the paradigm experiment. Our experiment was less accurate at identifying pairs of negative and neutral expressions, such as fear and surprise, in a limited set of two expressions. Facial expression recognition is mediated by a distributed neural system in humans that involves multiple bilateral regions (Haxby et al., 2000). The discrimination of facial expressions may be reflected in brain activity patterns. For example, Vrticka et al. (2014) considered fear as a negatively valenced surprise. We hypothesized that the brain regions that govern the expression of these two facial expressions may come from the same region, which also increases the difficulty of expression recognition and leads to a decrease in accuracy. In addition, many earlier studies have used small samples (Chiu et al., 2018; Yılmaz et al., 2019), or selected several facial expressions for research (Milders et al., 2010; Lawlor-Savage et al., 2014), as well as experiments to study the perception of different expression tensions (Branco et al., 2017). Conversely, although our sample size was small, each subject was selected from two limited facial expressions and a total of 300 times were recognized for six facial expressions of 10 models, which significantly increased the stability and accuracy of the experiment. Moreover, as in several earlier studies, we did not consider the length of stimulus presentation as a variable in itself and only varied the duration to avoid habituation (Jerram et al., 2013; Gohier et al., 2014).

The second finding is that the decline in the facial expression recognition ability of patients with depression is related to the decline of cognitive function, especially for negative facial expressions. The recognition of negative facial expressions is mainly related to processing speed, reasoning, and problem-solving ability, which is consistent with earlier studies. Major depressive disorder is frequently associated with cognitive impairment. Earlier research has shown that depression is related to executive function, memory, and processing speed (Nebes et al., 2000; Butters et al., 2004; Chen et al., 2015). The results of a meta-analysis showed that patients with depression have various forms of cognitive decline, including attention, processing speed, executive function, and memory (Lim et al., 2013). In clinical practice, further research is needed to improve the cognitive function of patients by improving their processing speed, reasoning ability, and problem-solving ability. In addition, distinguishing depressed patients from the normal control group by recognizing facial expressions is the next step in our research.

Emotional dysregulation is a key clinical feature of depression. An increasing number of studies have shown that emotional processing and emotional regulation in patients with affective disorders are impaired, and some of them have potential neurocognitive dysfunction (Assion et al., 2010). Frank et al. (2014) believed that successful emotion regulation often implies prefrontal control over emotional reactivity associated with amygdala responses. The present functional magnetic resonance imaging shows that, when compared with healthy people, those suffering from depression have greater subcortical limbic activity when recognizing positive and negative facial expressions (Almeida et al., 2009). Studies have also found that the amygdala is involved in the recognition of not only fear but also other expressions such as happiness and sadness (Juruena et al., 2010). The ventromedial prefrontal cortex (vmPFC) is related to various social, cognitive, and affective functions that are commonly disrupted in mental illness (Hiser and Koenigs, 2018). The current research suggests that the vmPFC regulates the fear response by inhibiting the amygdala (Quirk et al., 2003; Rosenkranz et al., 2003). Therefore, we speculated that patients with depression may differ from healthy people in brain areas such as the amygdala, the hippocampus, and the vmPFC.

The study on the structure and function of the brain in patients with depression has suggested some possible explanations for the link between cognitive decline and depression. The frontoparietal network is involved in the top–down regulation of attention and emotion, while the default network and the dorsal attention network are involved in internal and external attention, respectively (Nani et al., 2019). The dysregulation of the dorsolateral prefrontal cortex, which is associated with executive function, has been reported in the functional neuroimaging studies of depression during executive function. By using the meta-analysis, Kaiser et al. (2015) found that in the frontal network of depressed patients, there was low connectivity between the brain regions related to the cognitive control of attention and emotion regulation and between the frontoparietal and the parietal regions. Earlier research has suggested that WM, processing speed, and nonverbal memory capabilities are the indispensable components of emotional perception (Phillips et al., 2008; Mathersul et al., 2009). When attention, problem-solving ability, and speed of patients with depression decrease, the error rate of facial expression recognition increases. This implies that the decline in cognition in patients with depression may be caused by an imbalance between the brain networks. Thus, it is significant for the clinical diagnosis and treatment of depression to explore the abnormal brain structure and the mechanism of depression through the recognition of facial expressions in patients with depression.

This study has several limitations. First, whether the decline in facial expression recognition is caused by depression or cognitive decline remains an open question. Second, although the credibility of the experiment was high, the sample size was small. The neural mechanisms of facial expression recognition in patients with depression will be the next step in our study.

## Conclusion

Since the cognitive function is impaired in patients with depression, the ability to recognize negative facial expressions declines, which is mainly reflected in processing speed, reasoning, problem-solving, and memory and is caused by the imbalance between the brain networks.

## Data Availability Statement

The original contributions presented in the study are included in the article/supplementary material, further inquiries can be directed to the corresponding authors.

## Ethics Statement

The studies involving human participants were reviewed and approved by Ethics Committee of Beijing Huilongguan Hospital and the Ethics Committee of Zhumadian Mental Hospital. The patients/participants provided their written informed consent to participate in this study.

## Author Contributions

WZ and YF developed the concept and design of this study. MR and GH performed the experiments and analyzed the data. CN, LP, LS, TS, TY, SJ, and TL restructured, polished, and revised the manuscript. All authors contributed to the article and approved the submitted version.

### Conflict of Interest

The authors declare that the research was conducted in the absence of any commercial or financial relationships that could be construed as a potential conflict of interest.

## References

[ref300] Almeida J. R. C. D. Versace A. Mechelli A. Hassel S. Quevedo K. Kupfer D. J.. (2009). Abnormal amygdala-prefrontal effective connectivity to happy faces differentiates bipolar from major depression. Biol. Psychiatry 6, 451–459. 10.1016/j.biopsych.2009.03.024, PMID: 19450794 PMC2740996

[ref2] Assion H. J. Wolf F. Brüne M. (2010). P01-190—Theory of mind and neurocognitive functioning in patients with bipolar disorder. Bipolar Disord. 26:191. 10.1016/S0924-9338(11)71901-0 20882710

[ref3] Barrett L. F. Mesquita B. Gendron M. (2011). Context in emotion perception. Curr. Dir. Psychol. Sci. 20, 286–290. 10.1177/0963721411422522

[ref4] Beck A. T. (1967). Depression: clinical, experimental, and theoretical aspects. JAMA 203, 1144–1145. 10.1001/jama.1968.03140130056023

[ref5] Behere R. V. (2015). Facial emotion recognition deficits: the new face of schizophrenia. Indian J. Psychiatry 57, 229–235. 10.4103/0019-5545.166641, PMID: 26600574 PMC4623639

[ref6] Bone J. K. Lewis G. Button K. S. Duffy L. Lewis G. (2019). Variation in recognition of happy and sad facial expressions and self-reported depressive symptom severity: a prospective cohort study. J. Affect. Disord. 257, 461–469. 10.1016/j.jad.2019.06.025, PMID: 31310908

[ref7] Branco L. D. Cotrena C. Ponsoni A. Salvador-Silva R. Sjl V. Fonseca R. P. (2017). Identification and perceived intensity of facial expressions of emotion in bipolar disorder and major depression. Arch. Clin. Neuropsychol. 33, 1–11. 10.1093/arclin/acx080, PMID: 28961928

[ref8] Butters M. A. Whyte E. M. Nebes R. D. Begley A. E. Dew M. A. Mulsant B. H.. (2004). The nature and determinants of neuropsychological functioning in late-life depression. Arch. Gen. Psychiatry 61, 587–595. 10.1001/archpsyc.61.6.587, PMID: 15184238

[ref9] Chen J. Zhang Y. Wei D. Wu X. Fu Q. Xu F.. (2015). Neurophysiological handover from MMN to P3a in first-episode and recurrent major depression. J. Affect. Disord. 174, 173–179. 10.1016/j.jad.2014.11.049, PMID: 25499685

[ref10] Chiu I. Piguet O. Diehl-Schmid J. Riedl L. Beck J. Leyhe T.. (2018). Facial emotion recognition performance differentiates between behavioral variant frontotemporal dementia and major depressive disorder. J. Clin. Psychiatry 79:19. 10.4088/JCP.16m11342, PMID: 29360290

[ref11] Cotter J. Granger K. Backx R. Hobbs M. Looi C. Y. Barnett J. H. (2018). Social cognitive dysfunction as a clinical marker: a systematic review of meta-analyses across 30 clinical conditions. Neurosci. Biobehav. Rev. 84, 92–99. 10.1016/j.neubiorev.2017.11.014, PMID: 29175518

[ref12] Frank D. W. Dewitt M. Hudgens-Haney M. Schaeffer D. J. Ball B. H. Schwarz N. F.. (2014). Emotion regulation: quantitative meta-analysis of functional activation and deactivation. Neurosci. Biobehav. Rev. 45, 202–211. 10.1016/j.neubiorev.2014.06.010, PMID: 24984244

[ref13] Goghari V. M. Sponheim S. R. (2013). More pronounced deficits in facial emotion recognition for schizophrenia than bipolar disorder. Compr. Psychiatry 54, 388–397. 10.1016/j.comppsych.2012.10.012, PMID: 23218816 PMC3600398

[ref3000] Gohier B. Senior C. Radua J. El-Hage W. Reichenberg A. Proitsi P.. (2014). Genetic modulation of the response bias towards facial displays of anger and happiness. Eur. Psychiat. 29, 197–202. 10.1016/j.eurpsy.2013.03.003, PMID: 23769682

[ref14] Gollan J. K. McCloskey M. Hoxha D. Coccaro E. F. Watson D. (2010). How do depressed and healthy adults interpret nuanced facial expressions? J. Abnorm. Psychol. 119, 804–810. 10.1037/a0020234, PMID: 20939654 PMC3805828

[ref15] Gur R. C. Erwin R. J. Gur R. E. Zwil A. S. Kraemer H. C. (1992). Facial emotion discrimination: II. Behavioral findings in depression. Psychiatry Res. 42, 241–251. 10.1016/0165-1781(92)90116-K, PMID: 1496056

[ref16] Haxby J. V. Hoffman E. A. Gobbini M. I. (2000). The distributed human neural system for face perception. Trends Cogn. Sci. 4, 223–233. 10.1016/S1364-6613(00)01482-0, PMID: 10827445

[ref17] Hayashi S. Terada S. Takenoshita S. Kawano Y. Yabe M. Imai N.. (2021). Facial expression recognition in mild cognitive impairment and dementia: is the preservation of happiness recognition hypothesis true? Psychogeriatrics 21, 54–61. 10.1111/psyg.12622, PMID: 33191622

[ref18] Heck A. Hock A. White H. Jubran R. Bhatt R. S. (2017). Further evidence of early development of attention to dynamic facial emotions: reply to Grossmann and Jessen. J. Exp. Child Psychol. 153, 155–162. 10.1016/j.jecp.2016.08.006, PMID: 27686256 PMC5191505

[ref19] Hiser J. Koenigs M. (2018). The multifaceted role of the ventromedial prefrontal cortex in emotion, decision making, social cognition, and psychopathology. Biol. Psychiatry 83, 638–647. 10.1016/j.biopsych.2017.10.030, PMID: 29275839 PMC5862740

[ref20] Jerram M. Lee A. Negreira A. Gansler D. (2013). The neural correlates of the dominance dimension of emotion. Psychiatry Res. 221, 135–141. 10.1016/j.pscychresns.2013.11.007, PMID: 24359970

[ref21] Juruena M. F. Glampietro V. P. Smith S. D. Surguladze S. A. Dalton J. A. Benson P. J.. (2010). Amygdala activation to masked happy facial expressions. J. Int. Neuropsychol. Soc. 16, 383–387. 10.1017/S1355617709991172, PMID: 19958569

[ref22] Kaiser R. H. Andrews-Hanna J. R. Wager T. D. Pizzagalli D. A. (2015). Large-scale network dysfunction in major depressive disorder: a meta-analysis of resting-state functional connectivity. JAMA Psychiat. 72, 603–611. 10.1001/jamapsychiatry.2015.0071, PMID: 25785575 PMC4456260

[ref23] Lawlor-Savage L. Sponheim S. R. Goghari V. M. (2014). Impaired recognition of happy facial expressions in bipolar disorder. Acta Neuropsychiatr. 26, 253–259. 10.1017/neu.2014.6, PMID: 25142295

[ref24] Lee S. Liu C. Kuo C. Hsueh I. Hsieh C. (2020). Sensitivity and specificity of a facial emotion recognition test in classifying patients with schizophrenia. J. Affect. Disord. 275, 224–229. 10.1016/j.jad.2020.07.003, PMID: 32734912

[ref25] Lee J. Mathews A. Shergill S. Yiend J. (2016). Magnitude of negative interpretation bias depends on severity of depression. Behav. Res. Ther. 83, 26–34. 10.1016/j.brat.2016.05.007, PMID: 27262590

[ref26] Leib S. (2019). Shedding light on the interconnected relationship between depressive symptoms, facial emotion recognition, and working memory. ProQuest Dissertations Publishing.

[ref27] Leppänen J. M. Milders M. Bell J. S. Terriere E. Hietanen J. K. (2004). Depression biases the recognition of emotionally neutral faces. Psychiatry Res. 128, 123–133. 10.1016/j.psychres.2004.05.020, PMID: 15488955

[ref28] Lim J. Oh I. K. Han C. Huh Y. J. Jung I. Patkar A. A.. (2013). Sensitivity of cognitive tests in four cognitive domains in discriminating MDD patients from healthy controls: a meta-analysis. Int. Psychogeriatr. 25, 1543–1557. 10.1017/S1041610213000689, PMID: 23725644

[ref29] Macmillan N. A. Creelman C. D. (2005). Detection Theory: A User’s Guide. 2nd Edn. Vol. 8. New York: Psychology Press, 110–117.

[ref1600] Mathersul D. Palmer D. M. Gur R. C. Gur R. E. Cooper N. Gordon E.. (2009). Explicit identification and implicit recognition of facial emotions: II. Core domains and relationships with general cognition. J. Clin. Exp. Neuropsycol. 31, 278–291. 10.1080/13803390802043619, PMID: 18720178

[ref31] Mathews A. MacLeod C. (2005). Cognitive vulnerability to emotional disorders. Annu. Rev. Clin. Psychol. 1, 167–195. 10.1146/annurev.clinpsy.1.102803.143916, PMID: 17716086

[ref32] Milders M. Bell S. Platt J. Serrano R. Runcie O. (2010). Stable expression recognition abnormalities in unipolar depression. Psychiatry Res. 179, 38–42. 10.1016/j.psychres.2009.05.015, PMID: 20478626

[ref33] Miskowiak K. W. Kjærstad H. L. Meluken I. Petersen J. Z. Carvalho A. F. (2016). The search for neuroimaging and cognitive endophenotypes: a critical systematic review of studies involving unaffected first-degree relatives of individuals with bipolar disorder. Neurosci. Biobehav. Rev. 73, 1–22. 10.1016/j.neubiorev.2016.12.011, PMID: 27979650

[ref34] Nani A. Manuello J. Mancuso L. Liloia D. Costa T. Cauda F. (2019). The neural correlates of consciousness and attention: two sister processes of the brain. Front. Neurosci. 13:1169. 10.3389/fnins.2019.01169, PMID: 31749675 PMC6842945

[ref35] Nebes R. D. Butters M. A. Mulsant B. H. Pollock B. G. Zmuda M. D. Houck P. R.. (2000). Decreased working memory and processing speed mediate cognitive impairment in geriatric depression. Psychol. Med. 30, 679–691. 10.1017/S0033291799001968, PMID: 10883722

[ref1500] Phillips L. H. Channon S. Tunstall M. Hedenstrom A. Lyons K. (2008). The role of working memory in decoding emotions. Emotion 8, 184–191. 10.1037/1528-3542.8.2.184, PMID: 18410192

[ref37] Phillips L. H. Scott C. Henry J. D. Mowat D. Bell J. S. Blanchard-Fields F. (2010). Emotion perception in Alzheimer’s disease and mood disorder in old age. Psychol. Aging 25, 38–47. 10.1037/a0017369, PMID: 20230126

[ref700] Quirk G. J. Likhtik E. Pelletier J. G. Paré D. (2003). Stimulation of medial prefrontal cortex decreases the responsiveness of central amygdala output neurons. J. Neurosci. 23, 8800–8807. 10.1523/JNEUROSCI.23-25-08800.2003, PMID: 14507980 PMC6740415

[ref39] Rao D. Xu G. Lu Z. Liang H. Lin K. Tang M. (2019). Comparative study of cognitive function between treatment-resistant depressive patients and first-episode depressive patients. Neuropsychiatr. Dis. Treat. 15, 3411–3417. 10.2147/ndt.s226405, PMID: 31849475 PMC6910859

[ref40] Roiser J. P. Elliott R. Sahakian B. J. (2012). Cognitive mechanisms of treatment in depression. Neuropsychopharmacology 37, 117–136. 10.1038/npp.2011.183, PMID: 21976044 PMC3238070

[ref800] Rosenkranz J. A. Moore H. Grace A. A. (2003). The prefrontal cortex regulates lateral amygdala neuronal plasticity and responses to previously conditioned stimuli. J. Neurosci. 23, 11054–11064. 10.1523/jneurosci.23-35-11054.2003, PMID: 14657162 PMC6741051

[ref42] Sheardova K. Laczó J. Vyhnalek M. Andel R. Mokrisova I. Vlcek K.. (2014). Famous landmark identification in amnestic mild cognitive impairment and Alzheimer’s disease. PLoS One 9:e105623. 10.1371/journal.pone.0105623, PMID: 25144755 PMC4140812

[ref43] Vechetova G. Pastrnak M. Sevcikova M. Bartoskova M. Preiss M. (2019). Cognitive functions in depression—current perspectives. Cesk. Psychol. 63, 562–575.

[ref44] Vidal-Ribas P. Brotman M. A. Salum G. A. Kaiser A. Meffert L. Pine D. S.. (2018). Deficits in emotion recognition are associated with depressive symptoms in youth with disruptive mood dysregulation disorder. Depress. Anxiety 35, 1207–1217. 10.1002/da.22810, PMID: 30004611 PMC9719110

[ref45] Vrticka P. Lordier L. Bediou B. Sander D. DeSteno D. (2014). Human amygdala response to dynamic facial expressions of positive and negative surprise. Emotion 14, 161–169. 10.1037/a0034619, PMID: 24219397

[ref46] Wang Y. Su Y. Fang P. Zhou Q. (2011). Facial expression recognition: can preschoolers with cochlear implants and hearing aids catch it? Res. Dev. Disabil. 32, 2583–2588. 10.1016/j.ridd.2011.06.019, PMID: 21807479

[ref47] Wiechetek Ostos M. Schenk F. Baenziger T. von Gunten A. (2011). An exploratory study on facial emotion recognition capacity in beginning Alzheimer’s disease. Eur. Neurol. 65, 361–367. 10.1159/000327979, PMID: 21625142

[ref48] Woo Y. S. Rosenblat J. D. Kakar R. Bahk W. McIntyre R. S. (2016). Cognitive deficits as a mediator of poor occupational function in remitted major depressive disorder patients. Clin. Psychopharmacol. Neurosci. 14, 1–16. 10.9758/cpn.2016.14.1.1, PMID: 26792035 PMC4730927

[ref49] Yang C. Zhang T. Li Z. Heeramun-Aubeeluck A. Liu N. Huang N.. (2015). The relationship between facial emotion recognition and executive functions in first-episode patients with schizophrenia and their siblings. BMC Psychiatry 15:241. 10.1186/s12888-015-0618-3, PMID: 26449211 PMC4599651

[ref50] Yılmaz O. Mırçık A. B. Kunduz M. Çombaş M. Öztürk A. Deveci E.. (2019). Effects of cognitive behavioral therapy, existential psychotherapy and supportive counselling on facial emotion recognition among patients with mild or moderate depression. Psychiatry Investig. 16, 491–503. 10.30773/pi.2019.03.14, PMID: 31352731 PMC6664217

[ref51] Zwick J. C. Wolkenstein L. (2016). Facial emotion recognition, theory of mind and the role of facial mimicry in depression. J. Affect. Disord. 210, 90–99. 10.1016/j.jad.2016.12.022, PMID: 28024224

